# Explaining the Consequences of Imprisonment for Union Formation and Dissolution in Denmark

**DOI:** 10.1002/pam.21933

**Published:** 2016-07-05

**Authors:** Peter Fallesen, Lars H. Andersen

## Abstract

Crime and subsequent imprisonment reduces men's chances on the marriage market and increases their divorce risk, but existing research, with a few notable exceptions, is silent about the underlying mechanisms driving these effects. This article studies the effect of home confinement under electronic monitoring as a noncustodial alternative to imprisonment on the risk of relationship dissolution and being single, thereby distinguishing between effects of incarceration and of committing crime. We study a policy that expanded the use of electronic monitoring to address nonrandom selection into electronic monitoring instead of in prison. Results from a sample of 4,522 men show that home confinement under electronic monitoring significantly and persistently lowers the risk both of being single and of becoming single during the first five years following conviction. The results show that one of the tools that could promote decarceration trends also secures better relationship outcomes of convicted men.

## INTRODUCTION

Imprisonment impairs the future labor market chances of offenders and contributes to social inequality (e.g., Western, 2006), and recent research finds that imprisonment reduces men's chances on the marriage market (e.g., Svarer, 2011) and increases their divorce risk (e.g., Apel et al., 2010; Lopoo & Western, 2005; Massoglia, Remster, & King, 2011; Siennick, Stewart, & Staff, 2014). Importantly, the negative consequences of imprisonment for romantic relationships correlate both with sentence length (Massoglia, Remster, & King, 2011) and with the severity of the crime (Apel et al., 2010). Also, recent research finds immediate and persistent consequences for cohabitation as well as long‐term consequences for marriage chances of even short spells of jail or prison incarceration (Apel, 2016).

But empirical knowledge on the negative consequences of imprisonment for romantic relationships is still incomplete. We do not know, for example, whether imprisonment directly affects offenders’ potential as romantic partners, or whether other characteristics that make offenders less suitable as romantic partners even in the absence of conviction and imprisonment confound the negative association between imprisonment and romantic relationships. Also, even though incarceration is not a discrete event and offenders receive different sentence lengths, we currently do not know whether the negative association between imprisonment and romantic relationships is driven by incarceration as a discrete event or by length of incarceration causing spousal separation and human capital depletion (see, however, Massoglia, Remster, & King, 2011).

In this study, we address these issues. We do so by providing a strong causal test of the effect of imprisonment relative to electronic monitoring on union formation and dissolution and on singlehood, and by examining these effects across sentence length. We outline a number of mechanisms through which imprisonment could affect union formation and dissolution, mechanisms that electronic monitoring (in the specific way it was implemented in Denmark) could help to mitigate. To minimize the risk of confounding factors and to obtain uncontaminated effect estimates, we rely on data from before and after a policy reform that expanded the use of home confinement under electronic monitoring as a noncustodial alternative to imprisonment in Denmark in 2008. Also, to further minimize the risk of confounding factors we exploit the panel structure of our data and include in our statistical models individual fixed effects. These individual fixed effects control for time‐invariant unobserved factors that affect both selection into home confinement under electronic monitoring and into romantic relationships, selection issues that could otherwise contaminate our effect estimates. We use Danish register data on *N* = 4,522 men who were older than 25 years of age and who were sentenced to imprisonment for up to three months for non‐traffic‐related offences in the period 2006 to 2009. We link the crime data with official housing registers to obtain relationship status. These data then contain the entire population of convicted offenders targeted by the policy reform in 2008, of whom some experienced imprisonment and others served their prison sentence at home under electronic monitoring. The reform introduced the use of electronic monitoring among the group, causing more than 33 percent of all offenders in the sample to serve with electronic monitoring after the reform compared to none before.

Previous research has studied how alternatives to imprisonment affect, for example, criminal recidivism (e.g., Di Tella & Schargrodsky, 2013), labor market attachment (e.g., Andersen & Andersen, 2014), educational achievement (Larsen, 2016), and children's foster care placements (Andersen & Wildeman, 2014). Our study is the first to isolate the effect of a noncustodial alternative to imprisonment, such as electronic monitoring, on relationship dissolution and on singlehood.

We find that electronic monitoring significantly and substantially decreases the risk of relationship dissolution and singlehood during at least the first five years following conviction. For the entire sample, intention‐to‐treat (ITT) estimates show that the introduction of electronic monitoring on average decreased the risk of being single by more than 4 percentage points after the conviction, compared to the pre‐reform group. For men who were in a relationship when convicted, the reform decreased the risk of being single by 9 percentage points. For men who were not in a relationship when convicted, the reduction was smaller, yet still statistically significant, at 3 percentage points. Importantly, these estimates are based on a research design that offers variation in electronic monitoring that is plausibly exogenous to individual characteristics, and the estimates may thus be interpreted as causal effects. And, indeed, because we also control for unobserved time‐invariant factors that affect selection into both electronic monitoring and romantic relationships (individual‐level fixed effects), the causal nature of the estimates is further enhanced. Examining the effect across sentence length shows that the effects do not differ by shorter or longer sentences when controlling for such unobserved time‐invariant differences between offenders with different sentence lengths, which stresses the importance of applying individual‐level fixed effects.

## PARTNERSHIP, INCARCERATION, AND DIVORCE

Research has linked imprisonment to increased divorce risks (Apel et al., 2010) and to immediate disruptions of residential relationships (Apel, 2016), just as research has shown that it is harder for men marked by a criminal record (and who have been imprisoned) than for nonconvicted men to find a partner and marry (Svarer, 2011; see, however, King, Massoglia, & MacMillan, 2007; Lopoo & Western, 2005). Nevertheless, it remains empirically unanswered what drives these negative associations between imprisonment and romantic relationships. Many processes associated with imprisonment could lead to poorer marriage market opportunities and to elevated risks of relationship dissolution and singlehood following release. In what follows, we focus on the social stigma of imprisonment; the depletion of human capital during and after imprisonment and imprisonment as an indicator of poor match quality; and the separation from significant others during imprisonment—and on how these mechanisms could vary with sentence length. Then we discuss electronic monitoring as a noncustodial alternative to imprisonment and relate this to the outlined mechanisms.

### Social Stigma

One explanation of the link between imprisonment and romantic relationships is that criminal conviction and imprisonment place severe social stigma on offenders (e.g., Braman, 2004). Social stigma is a process by which people infer unobserved negative traits about a person from one observed characteristic or life event, such as imprisonment (Goffman, 1963). In this way, imprisonment may signal, correctly or incorrectly, that the imprisoned person is an unfit romantic partner, which could matter for his or her chances on the marriage market as well as for his or her divorce risk.

Research has also demonstrated that imprisonment places social stigma on people who are in a romantic relationship with an offender (e.g., Comfort, 2008). In much the same way as when a criminal offender faces social stigma, a partner's imprisonment may signal unobserved negative traits about the nonimprisoned romantic partner. Staying in the relationship could very well create strain from spillovers of stigma from the offender to the nonimprisoned partner, which could lead to an increased divorce risk. The social stigma of incarceration could be structured as a binary marker and depend on whether or not a person experienced incarceration (or, is known to have), or stigma could be a qualitative marker and correlate with length of incarceration (longer sentences leading to more stigma).

### Human Capital Depletion and Indication of Poor Match Quality

Imprisonment depletes human capital by introducing spells of nonemployment and a loss of acquired skills during imprisonment (Western, Kling, & Weiman, 2001). Such depletion could be part of a process of cumulative disadvantage lasting beyond the imprisonment if followed by a period of nonstandard employment, misemployment, or underemployment (Pedulla, 2016), further decreasing human capital. Recent empirical research, which uses empirical setups similar to what we use in this study, compares labor market outcomes among imprisoned people and people who experience noncustodial alternatives to imprisonment, such as community service (Andersen, 2015) or electronic monitoring (Andersen & Andersen, 2014). Results from these studies show that imprisonment causes worse labor market outcomes following release than noncustodial alternatives do. Thus, imprisonment impairs offenders’ life chances, and could make them less valuable on the marriage market.

This mechanism not only complicates single men's search for a partner (Becker, 1973), but it also increases the relationship dissolution risk among offenders who are in romantic relationships or who are married (Becker, Landes, & Michael, 1977). In addition, offenders gain criminal capital during imprisonment through interactions with imprisoned peers, which likely correlates negatively with human capital sought by employers (Bayer, Hjalmarsson, & Pozen, 2009). This could also make them less attractive romantic partners. And, indeed, studies show that criminal recidivism is lower among electronically monitored offenders than among imprisoned offenders (e.g., Di Tella & Schargrodsky, 2013).

Positive things could happen in prison, too, and imprisonment is not just a period of human capital depletion and bad company. There are labor market programs and training in prison, which could increase offenders’ human capital. This is especially true in the Danish context, a point we return to later. In addition, offenders with drug or alcohol problems may be subject to professional treatment during imprisonment—again something their spouses might appreciate and that could lead to lower rates of relationship dissolutions following release.

### Separation

In addition to social stigma and human capital depletion, which impairs the future life chances of all imprisoned people, imprisonment places an additional burden on people who have a romantic partner in the community. Namely, imprisonment also implies the physical separation of the romantic partners. All prisoners are separated from family members and their loved ones, yet being separated from a romantic partner implies being separated from a very intimate relationship, one that offers a pooling of resources, strong emotional ties, and safe and regular sex. Spousal separation, which means not having these very intimate ties, may lead to spousal estrangement (Massoglia, Remster, & King, 2011) and to elevated household strain due to a decrease in the offender's financial and emotional support to their partner and the household during imprisonment (e.g., Apel et al., 2010). In addition, recent research finds that women report decreased relationship quality during the years after a partner's incarceration (Turney, 2015).

The physical separation of partners during imprisonment likely affects divorce risks. Massoglia, Remster, and King (2011) identify three ways how the effect might play out. First, physical separation could lower relationship satisfaction, for example, by decreasing emotional interactions between the partners and by reducing available social support. Second, with one adult missing from the household, the nonincarcerated partner faces a much larger household workload and becomes the sole provider for a time. Third, people change, and due to the lack of day‐to‐day interactions between romantic partners, they may have a hard time reconciling old impressions with new ones following imprisonment, simply because they have not had the opportunity to experience these changes gradually. Overall, Massoglia, Remster, and King (2011) provide a strong test of the separation hypothesis, but due to data limitations, they have to assume that their correlational estimate is accurate despite the lack of exogenous variation.

### Sentence Length

Social stigma, the loss of human capital and poor match quality, as well as the separation of spouses, are all mechanisms that occur over time. It is thus likely that the impact of imprisonment on relationship dissolution and singlehood correlates with sentence length, simply because lengthier sentences leave more time for the outlined mechanisms to take effect. If so, we should expect to see stronger effects of imprisonment among offenders who serve comparatively long sentences. Yet, even brief contact with the criminal justice system may have devastating effects on families too. Apel (2016) finds that comparatively brief periods of jail or prison incarceration (median incarceration period of about one month) are immediately consequential for residential partnerships, and also that these brief incarcerations decrease people's chances on the marriage market in the longer run. From the literature on family dynamics, we know that family instability has substantial effects on family members, especially on children, over and above the mechanisms that select people into those families (Fomby & Cherlin, 2007). Thus, to the degree that even brief criminal justice contact exacerbates family instability, it could even be the case that shorter sentences are more damaging to families than longer sentences. Still, any correlation between sentence length and the mechanisms that drive the effect of imprisonment on relationship dissolution and singlehood could also be fully or partly caused by the selection of offenders into sentence lengths. Offenders who get longer sentences also have worse characteristics (observed as well as unobserved) than offenders who get shorter sentences, and these characteristics could ultimately produce worse relationship outcomes among offenders with longer sentences—not the long sentence in itself. If the latter is the case, we should expect to find similar results for shorter and longer sentences.

Sentence lengths for comparable cases did not change around the time of the reform we use for causal inference (Andersen & Andersen, 2014). This means that we may run analyses by sentence length to show whether offenders who get shorter or longer sentences suffer more or less from the mechanisms that tie imprisonment to relationship outcomes.

### Imprisonment in Denmark

Prison sentences in Denmark cover any sentence length (longer than seven days, which is the minimum sentence length in Denmark) handed down by a sentencing court. There is no distinction between jail incarceration and imprisonment, as there is in the United States, where sentences of more than nine months would typically send the offender to prison rather than jail. There are local arrest houses in Denmark, but these are used primarily for pre‐trial incarceration. Danish prisons are divided into low‐security (open) prisons and high‐security (closed) prisons. Offenders sentenced to less than five years, which includes all offenders in our sample, will typically serve their sentence in low‐security prisons. These low‐security prisons differ greatly from penal institutions in countries with less rehabilitative punishment ideologies, such as the United States, where harsh prison conditions are viewed as part of the punishment. Doors are unlocked, a fence rather than a wall surrounds the prison, and inmates are allowed to pursue pro‐social activities, like taking up education or employment, outside the prison.

These conditions of confinement could make it easier for prisoners to maintain contact with their loved ones, just as these conditions could make it easier to keep employment and pursue education during incarceration. Most prisons in Denmark are, however, located far from the larger cities (which is where jobs and education facilities are likely to be located), and these locations are not necessarily within their partner's everyday commuting distance. Thus, physical contact between partners does not occur often, also because all visits have to be reported and approved by the prison. According to a recent Danish survey among inmates (Lindstad, 2015), family contact during imprisonment is not easily accessible. Likewise, human capital outcomes of prisoners in Denmark are generally lower than what the above‐mentioned conditions of confinement could imply. On any given day, for example, around 100 out of the 1,300 prisoners in open prisons are enrolled in education outside the prison premises—and a total of around 20 percent of prisoners pursue education while incarcerated, mostly elementary schooling (Koudahl, 2010).

Once released from prison in Denmark, people face challenges of reintegrating into communities because they now have a criminal record. Criminal records in Denmark are sealed but employers may still request a job candidate's record. The sealing of criminal records implies that a man's (potential) spouse cannot check his criminal record without his consent. Thus, men may try to hide their incarceration or criminal justice contact from their (potential) spouse. Wives are probably not likely to remain ignorant of incarceration or electronic monitoring (especially because electronic monitoring requires the wife's formal acceptance). But incarceration for one or a few weeks could probably be disguised to a potential spouse as being away on holiday—and the criminal justice contact that occurred before the potential spouse was even met might not be brought to light at all. Thus, stigma effects of incarceration could be much lower in Denmark than in the United States, especially for men who were not in a relationship during incarceration.

#### Electronic Monitoring: The Danish Case

Electronic monitoring in Denmark is a way of serving a prison sentence. If offenders’ personal circumstances and their criminal cases adhere to formal preconditions, offenders can apply to serve their prison sentences at home under electronic monitoring. Some offenders apply to serve their sentence under electronic monitoring and receive permission to do so. Others either do not apply, are not permitted to do so, refuse to accept the conditions of electronic monitoring, or have their permission revoked because of technical violations.

Electronic monitoring implies that the offender is fitted with a GPS tracking device (anklet) and has to adhere to a strict time schedule. For example, even short deviations from the time schedule, such as coming home from work ten minutes late, will set off an alarm at the Danish Prison and Probation Service, which administers the electronic monitoring. The Prison and Probation Service then contacts the offender and demands an explanation of the delay. As a last resort, the Danish Prison and Probation Service can revoke the electronic monitoring permission and transfer the offender to prison. In addition to sticking to the strict organization of everyday life, the offender serving a prison sentence under electronic monitoring has to accept additional conditions, such as unscheduled spot‐tests for alcohol and drug use. Finally, electronically monitored offenders are required to participate in a crime prevention program (Danish Corrections Act, Law no. 367).

Eligibility to serve a prison sentence at home under electronic monitoring is not only obtained by accepting the conditions of electronic monitoring. Offenders must have a permanent address (e.g., not be living in a shelter for the homeless) and either be employed, participate in some form of active labor market program, or be enrolled in an education program. Unemployed offenders can fulfill this employment criterion by working at institutions appointed by the Prison and Probation Service. In addition, if the offender has cohabiting family members of age 18 or older, then these members must formally accept the electronic monitoring of the offender in the home. Employers must also formally accept having an employee working while he or she is electronically monitored. Last, only offenders who have not previously committed any serious crime—defined as a crime punishable by more than a fine—within two years prior to the conviction are eligible for serving a prison sentence at home under electronic monitoring (Danish Corrections Act, Law no. 367).^1^

The electronic monitoring scheme in Denmark gradually expanded since it was first introduced in May 2005. First, traffic offenders could serve prison sentences shorter than three months in their own homes under electronic monitoring. In April 2006, the scheme extended to include all offenders younger than 25 years of age, regardless of the offense type (still with a maximum sentence length of three months). In June 2008, the policy reform that we exploit in our analyses removed the age requirement. Offenders of all ages and regardless of offense type could now apply for electronic monitoring if their prison sentence did not exceed three months.^2^ Last, in 2010 and 2013 the maximum sentence length requirement of three months was expanded to five and six months, respectively, and the reform also abandoned the requirement of no serious crimes within two years prior to the conviction.

**Figure 1:**
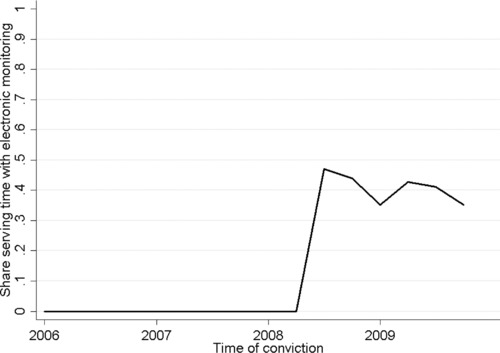
Share Completing Their Prison Sentence at Home Under Electronic Monitoring Among Men Targeted by the Reform in June 2008. *Notes*: Men older than 25 years of age, convicted of nontraffic offenses and with sentence length shorter than or equal to three months. To formally demonstrate that the reform did indeed significantly affect the likelihood of serving time with electronic monitoring, even net of covariates, we regressed electronic monitoring on the reform dummy and on offender covariates. Results, available in Table A1 in Supporting Information Appendix A, show that in all samples the 2008 reform had a substantial impact on the chance for serving a prison sentence at home under electronic monitoring rather than in prison, net of covariates. The reform increased the likelihood of serving a sentence under electronic monitoring by 33.9 (Full Sample), 44.0 (Relationship Sample), and 30.0 (No Relationship Sample) percentage points, all significant at the 0.001 level. The statistical relevance of the 2008 reform as a provider of change in the chance of serving a sentence at home under electronic monitoring is high, with *F*‐values of 1411.79 (Full Sample), 583.92 (Relationship Sample), and 855.04 (No Relationship Sample). All appendices are available at the end of this article as it appears in JPAM online. Go to the publisher's website and use the search engine to locate the article at http://onlinelibrary.wiley.com. *Source*: Own calculations on data from Statistics Denmark.

Figure 1 shows the sharp increase in the share of men targeted by the 2008 reform, which we use in our analyses, who completed their prison sentence at home under electronic monitoring. We use this reform in our analyses because it targets older offenders, of whom a comparatively large share is in a relationship upon conviction.^3^ The men in the figure are all 25 years of age or older, they are convicted of nontraffic offenses, and they received a prison sentence that was shorter than or equal to three months.^4^ The figure shows that before the 2008 reform all these men served their prison sentence in prison, while the same is only true for around 65 percent after the reform.

The maximum sentence length requirement of three months (today: six months) could seem to aim electronic monitoring at a very small group of offenders who receive such short prison sentences. However, sentences in Denmark are generally that short, and around two thirds of all prison sentences in Denmark are three months or shorter (Danish Prison and Probation Service, 2013). Offenses include violent offenses, minor drug offenses, and theft and property crimes. Thus, electronic monitoring is a noncustodial alternative to the majority of prison sentences in Denmark, and results in this study concern a nonnegligible share of offenders. Figure A1 in the Supporting Information Appendix A shows a histogram of sentence lengths for our sample.^5^ The median sentence length is 40 days.

### Electronic Monitoring and Romantic Relationships

Electronic monitoring in Denmark restricts the offender's daily movements and his or her social life outside the home, while at the same time requiring him or her to hold down regular employment or education and remain (physically) present in the family home while serving the prison sentence. Electronically monitored offenders also are required to follow a crime prevention program at the Danish Prison and Probation Service. The design and implementation of home confinement under electronic monitoring in Denmark could thus ameliorate some of the negative consequences of imprisonment on romantic relationships—not necessarily because the offender is wearing an anklet and is being electronically monitored, but also because all these other treatments occur simultaneously. Home confinement under electronic monitoring is, in this sense, a specific treatment package implemented in a specific way attuned to the Danish context, something we return to in the Discussion section. The anklet itself, at least primarily, is simply a technological tool that enables home confinement and secures compliance with the treatment package—or at least sets off an alert when the conditions of that treatment package are breached (DeMichele, 2014). When referring to electronic monitoring in this paper, it denotes a treatment package that consists of each of these elements.

For people who are not in a relationship, the main difference between imprisonment and electronic monitoring pertains to whether or not the social stigma of imprisonment exceeds the social stigma of serving a prison sentence at home under electronic monitoring, and whether or not human capital suffers more from imprisonment than from electronic monitoring. Empirical evidence that distinguishes between these two mechanisms is rare. But even though qualitative studies have found that wearing a visible GPS tracker produces public shame (e.g., Martin, Hanrahan, & Bowers, 2009), existing studies argue that other people are likely to view imprisonment as more stigmatizing (e.g., Western, Kling, & Weiman, 2001). Also, serving a prison sentence at home under electronic monitoring reduces the offender's absence from society, making it easier for the offender to keep or at least search for a job or a partner. Thus, compared to single offenders who are imprisoned, single offenders who serve a prison sentence at home under electronic monitoring are likely to have better chances on the marriage market because they are actually on the market during confinement, because they face less social stigma, and because their human capital suffers less during their sentence.

For people who are in a relationship, a partner's incarceration, due to the theoretical mechanisms that we outlined above, can lead to changes in the costs and rewards of being in that particular relationship and can make alternative partners comparatively more appealing (Siennick, Stewart, & Staff, 2014). The normative social repercussions of leaving a romantic partner could also be much lower if the partner is imprisoned, and staying in the relationship might even, as mentioned, create strain from spillovers of stigma from the offender to the nonincarcerated partner (e.g., Comfort, 2008). Thus, if the nonincarcerated partner uses information about the new life situation to update his or her beliefs about the relationship's feasibility compared to outside options, imprisonment could lead to a higher risk of relationship dissolution. When the imprisonment stretches for longer periods it could even provide the nonimprisoned partner with more time for the strain of a changed life situation to permeate everyday life, leading to even higher divorce and dissolutions risks.

Qualitative research stresses that during electronic monitoring the offender's family members are essentially serving the sentence along with the offender and have to cope with the offender's frustrations over his or her own lack of autonomy (e.g., Payne, 2014). Yet the romantic partner of an offender serving a prison sentence at home under electronic monitoring may still rely on his or her partner to participate in housework, supply additional household income, and be emotionally and physically available. The partner also does not face strain derived from having an incarcerated (i.e., absent) partner (nor do they have to spend time visiting their incarcerated partner), just as electronic monitoring does not place the nonimprisoned partner in a situation where he or she can easily imagine his or her life without the (absent) partner. Thus, we expect that couples are less likely to grow apart if it is possible for them to remain together in the home and community during the prison sentence.

## METHOD AND DATA

In this study, we compare singlehood rates and relationship dissolution rates across offenders who (1) have all committed similar crimes, (2) were all convicted for these crimes, and (3) were all sentenced to similar prison sentences for these crimes—some of the offenders just served their prison sentence at home under electronic monitoring rather than in prison. Thus, we compare two offender groups who on average differ only in that one group experiences imprisonment whereas the other group serves their prison sentence at home under electronic monitoring, and any difference between their outcomes is attributable to the difference between their imprisonment and electronic monitoring experience. We focus only on male offenders, because female offenders constituted as little as 6 percent of all offenders included in the sample we consider.^6^

### Estimating the Impact of the Reform

The sharp increase in the use of electronic monitoring for offenders older than 25 years of age around the reform in 2008 provides us with a plausibly exogenous shock to these men's incarceration risks. The reform allows us to test the effects on singlehood and relationship dissolution of serving a prison sentence at home under electronic monitoring rather than in prison. As already discussed, individual and unobserved offender traits correlate with who applies for, receives permission to, and eventually completes their sentence under electronic monitoring in Denmark. The selection has two causes: (1) it is offenders themselves who apply for (and accept) electronic monitoring, and (2) the Danish Prison and Probation Service actively prioritizes between offenders. Thus, electronic monitoring is endogenous to offender traits.

But whether or not an offender was convicted before or after the 2008 reform represents a change in all offenders’ probability of serving their prison sentence at home under electronic monitoring, which is plausibly uncorrelated with offender traits. To enhance our claim that the 2008 reform represents a change that is uncorrelated with offender traits—a situation typically referred to as a natural experiment—the reader should recall that electronic monitoring in Denmark is not a sentence passed by a judge. Instead, it is a way of serving a prison sentence. Thus, eligibility to use electronic monitoring is determined not by a judge but by the Prison and Probation Service after the judge has sentenced the offender to imprisonment.

To evaluate how the introduction of electronic monitoring as an alternative to incarceration affected the risk of singlehood, we estimate the following reduced form differences‐in‐differences model:

(1) $$\begin{array}{ccc} Single_{it} & = & \sum_{t=2001}^{2014}I{\left(t\right)}_{t}+\sum_{y=-4}^{5}I{\left(Y\right)}_{it}+X_{it}\beta \\ & & +\;\psi Policy_{i}+\varphi Policy_{i}\times I{\left(y>0\right)}_{it}+\alpha_{i}+\varepsilon_{it} \end{array}$$

where $I{\left(t\right)}_{t}$ is a set of year dummies capturing changes in singlehood rates over time, $I{\left(Y\right)}_{it}$ is a set of dummies capturing trends in singlehood rates the years before and after an offender received his conviction, $X_{it}$ is a set of covariates, $Policy_{i}$ is an indicator of whether an offender received his conviction before or after the introduction of the policy, $Policy_{i}\times I{\left(y>0\right)}_{it}$ is an indicator capturing changes in singlehood trends post‐conviction for offenders convicted after the policy introduction, $\alpha_{i}$ is the individual‐specific constant (individual‐level fixed effects), and $\varepsilon_{it}$ is the error‐term. If we assume—this is the common trends assumption—that offenders convicted after the reform would have had identical singlehood risks as offenders convicted before the reform (absent the introduction of the reform), then φ captures the ITT effect of introducing electronic monitoring on the postconviction risk of singlehood. Again, notice that because we have panel data the individual‐level fixed effects, $\alpha_{i}$, allow us to control for time‐invariant unobserved characteristics that affect selection both into electronic monitoring and into romantic relationships, unobserved characteristics that could otherwise bias our effect estimates. To take into account that all individuals in the data contributed with several observations, one per year, all standard errors are robust.

We use three steps to analyze whether the assumptions of the model in equation (1) are breached. First, because the common trends assumption is fundamentally untestable (because we cannot observe how trends for the treated would have been, absent of the reform), we test whether the treatment and control group had similar trends up until they committed their crime. Second, we test beyond simple *t*‐test statistics, which only condition on treatment status, whether our sample is conditionally balanced across observable characteristics. We do this by regressing the reform dummy on all observable characteristics (measured at the beginning of the year of the crime) and on sentence length. Third, to test whether the reform truly caused the differences we observe in relationship rates across the reform, or if it is simply a result of an underlying change in the population's relationship patterns, we run a set of pseudo‐regressions. We reestimate equation (1), but shift the entire data window, including the reform date, one year in each direction at a monthly increment. For example, we run one estimation for the data period 2005 to 2008, with a pseudo‐reform date on July 1, 2007. Then, we shift the window month by month until we get the data period 2007 to 2010, with pseudo‐reform date on July 1, 2009. If changing relationship patterns in the population drive our main results, we would observe statistically significant pseudo‐reform effect even in data windows which overlap only a little with the true reform.

Using the ITT estimate and the share of offenders who served their sentence under electronic monitoring among those who had the possibility of doing so, ($P[EM_{i}=1|Policy_{i}=1]$), we can further calculate the average effect of electronic monitoring on singlehood among those who served their sentence under this noncustodial alternative (the average treatment effect of the treated [ATT]). We know from the reform text, as well as from Figure 1, that no one served time with electronic monitoring prior to the reform (i.e., $P[EM_{i}=1|Policy_{i}=0]=0$). This means that we have one‐sided noncompliance in whether or not offenders served time with electronic monitoring. Following Bloom (1984), we can then calculate the average treatment effect on the treated as the Wald estimator: $ATT=\frac{ITT}{P(EM_{i}=1|Policy_{i}=1)}$ (see also Angrist & Pischke, 2009, pp. 163–164). Thus, we can estimate both the effect of introducing the policy on offenders’ singlehood risk post conviction, as well as the average individual effect of serving a sentence with electronic monitoring among those who did serve their sentence with electronic monitoring. Again, because our statistical model controls for unobserved time‐invariant selection into both electronic monitoring and into romantic relationships (individual‐level fixed effects), the credibility of this statistical exercise seems reasonable.

We estimate three versions of equation (1) to measure the effect of serving a prison sentence at home under electronic monitoring on relationship outcomes. First, we estimate one model using all men in our sample, both those who were in a relationship and those who were single when convicted, to estimate the effect of electronic monitoring on singlehood. This model provides an estimate of the effect of the policy on becoming single (for men who were in a relationship upon conviction) and on staying single (for men who were single upon conviction) simultaneously. Second, because we, as discussed, expect electronic monitoring to be especially beneficial to offenders who were in a relationship upon conviction (because these offenders do not suffer from spousal separation during imprisonment), we estimate another model using only men who were in a relationship upon conviction (i.e., $Single_{it}=0|Y=0$). This model provides a causal estimate of the effect on relationship dissolution (i.e., becoming single). Third, we estimate a model using only men who were not in a relationship upon conviction (i.e., $Single_{it}=1|Y=0$). We do this to provide a causal estimate of the effect of imprisonment on the risk of staying single. Not part of our main analyses, we also estimate equation (1) only among men who were married when convicted. This is to see whether results differ by divorce and nonmarital relationship dissolution. The results do not differ substantially from each other, so we only touch upon the divorce results in passing.

### Data, Sample, and Variables

We use full population register data available from Statistics Denmark. In Denmark, all citizens receive a unique personal identification number when they are born (or, for immigrants, when they obtain citizenship), and this number identifies the person in various administrative regards throughout his or her life. Statistics Denmark collects these data, which cover wide arrays such as tax forms, education, housing, marital status, and criminal justice contacts, from administrative agencies, and makes them available for research purposes. The personal identification numbers allow researchers to merge individual information from the various registers, and Danish register data then essentially constitute an individual‐level panel of all Danish citizens and a wide range of their communications with administrative agencies (for a discussion of the merits of these register data, see Lyngstad & Skardhamar, 2011).

From the register data, we use a sample of 4,522 men who were older than 25 years of age when they were sentenced to imprisonment for less than three months for a nontraffic offense in the period 2006 to 2009. We exclude men who committed crimes in the four years leading up to their focal offense, to avoid spillover effect from earlier penal reforms. Although additional requirements, such as being employed and having a permanent address, as mentioned, had to be met to ensure eligibility for serving a prison sentence at home under electronic monitoring, the sample of 4,522 men corresponds to the raw sample targeted by the reform in 2008. We merge this sample with information from the Danish Prison and Probation Service on who served their prison sentence at home under electronic monitoring and who served their sentence in prison.

As the dependent variable, we define singlehood as being unmarried and not cohabiting with a partner for each of the four years following conviction. We measure relationship status at the beginning of each calendar year, and we follow Statistics Denmark's official definition of cohabitating couples, which is structured as follows. People are defined as being in a cohabiting relationship if they share an address (down to apartment level) and are either (1) married, (2) in a registered partnership, (3) have at least one joint child, or (4) are of opposite sex, not related, within a 15‐year age‐gap of each other, and are the only two adults living in the household. Thus, this definition of cohabiting couples excludes men sharing residence with female relatives, but it soaks up roommates and housemates who share residence but are in fact not in a romantic relationship, a limitation that challenges the conceptual interpretation of singlehood in our context. But, provided that we only analyze men who are older than 25 years of age, this limitation is perhaps not the most important one. At age 25, most people have left the education system—and room or house sharing is in fact rather uncommon outside student life. At the same time, age 25 is when we start to see high transition rates into relationships for Danish men (Bruze, Svarer, & Weiss, 2015).

Research from other countries, such as the United States, have tended to view marriage and cohabitation as distinct family formation events (e.g., Sassler, 2004). But in Denmark, as well as in other Nordic countries, there are compelling reasons for viewing cohabitation and marriage as a single unified category (e.g., Heuveline & Timberlake, 2004). Svarer (2004) and Bruze, Svarer, and Weiss (2015), using the same relationship definition and similar data as we do, show that cohabitation and later marriage are linked, and to some extent interchangeable, in Denmark. Thus, in our main analyses we do not analyze the effect of electronic monitoring on divorce, we analyze the effect of electronic monitoring on being single or dissolving a romantic relationship. But to ensure that our results are not simply driven by noise in our measure of singlehood, we also run separate results based only on whether an offender is married or not, results that we do not explicitly present but that are available from the authors upon request.

To distinguish between the effect of electronic monitoring on singlehood and on relationship dissolution, we run the main analyses both using the full sample and using subsamples defined by whether or not the men were in a relationship upon conviction. We refer to these samples as the Full Sample (4,522 men), Relationship Sample (1,247 men), and No Relationship Sample (3,275 men). As control variables we add age, years of education, offense type (theft, violence, or other offenses), and ethnic minority background. We measure these control variables at the time of conviction.

## FINDINGS

### Descriptive Results

Table 1 shows descriptive statistics of the Full Sample, Relationship Sample, and No Relationship Sample. The table shows that 13.9 percent of the Full Sample served their prison sentence at home under electronic monitoring. Among men convicted after the reform, 33.7 percent served with electronic monitoring. The difference in singlehood rates between pre‐ and postreform groups at the beginning of the year offenders committed their crime was only 0.001 percentage points. There are no significant or substantial differences in other observable characteristics across reform status, although the prereform group was slightly older (by 0.39 years).

**Table 1:** Descriptive statistics at year of conviction, by sample and reform date

	Full Sample			Relationship Sample			No Relationship Sample		
	Full	Reform = 0	Reform = 1	Full	Reform = 0	Reform = 1	Full	Reform = 0	Reform = 1
Single	0.724	0.724	0.725						
	(0.447)	(0.447)	(0.447)						
Reform = 1	0.411			0.411			0.411		
	(0.492)			(0.492)			(0.492)		
EM = 1	0.139	0.000	0.337***	0.180	0.000	0.439**	0.128	0.000	0.298
	(0.346)	(0.000)	(0.473)	(0.385)	(0.000)	(0.497)	(0.328)	(0.000)	(0.458)
Age	36.163	36.003	36.393	36.599	36.070	37.359**	35.998	35.978	36.026
	(8.320)	(8.291)	(8.357)	(8.410)	(8.225)	(8.621)	(8.280)	(8.318)	(8.229)
Education in years	10.573	10.545	10.612	10.890	10.860	10.934	10.452	10.426	10.490
	(2.325)	(2.338)	(2.307)	(2.450)	(2.400)	(2.523)	(2.264)	(2.303)	(2.208)
Theft	0.118	0.116	0.122	0.083	0.073	0.096	0.132	0.132	0.131
	(0.323)	(0.320)	(0.327)	(0.275)	(0.261)	(0.294)	(0.338)	(0.339)	(0.338)
Violence	0.573	0.585	0.556	0.654	0.676	0.621*	0.543	0.550	0.532
	(0.495)	(0.493)	(0.497)	(0.476)	(0.468)	(0.486)	(0.498)	(0.498)	(0.499)
Other crime	0.308	0.299	0.322	0.264	0.250	0.283	0.325	0.317	0.337
	(0.462)	(0.458)	(0.467)	(0.441)	(0.434)	(0.451)	(0.469)	(0.466)	(0.473)
Minority	0.165	0.164	0.166	0.190	0.192	0.188	0.155	0.153	0.158
	(0.371)	(0.370)	(0.372)	(0.393)	(0.394)	(0.391)	(0.362)	(0.360)	(0.365)
*N*	4522	2663	1859	1247	735	512	3275	1928	1347

*Notes*: Standard deviations are in parentheses. We test the H_0_ :E(X|Reform=0)=E(X|Reform=1) for all covariates X.

*p < 0.05; **p < 0.01; ***p < 0.001.

In the Relationship Sample, 18.0 percent completed their sentence at home under electronic monitoring, and again, all electronic monitoring occurred following the reform with an uptake of 43.9 percent of the postreform group serving with an electronic anklet. For the Relationship Sample, we see some significant differences in age and offence type across the reform date. Those convicted postreform were on average 1.4 years older and 5 percentage points less likely to have committed a violent crime. Age, however, does not predict whether an offender served their sentence with an electronic anklet or not, so we do not believe that the slightly older postreform group should be cause for concern (see Table A1 in Supporting Information Appendix A).^7^

In the No Relationship Sample, 12.8 percent completed their sentence at home under electronic monitoring, with a take‐up rate at 29.8 percent among those convicted after the reform. For this sample, there are no significant differences for any observable characteristic.

Additional calculations not shown here, but available upon request, show that among those who were permitted to serve their sentence at home under electronic monitoring, 9 percent in both samples failed to abide by the requirements and had their permission revoked. We include these men in the group of electronically monitored offenders, even though they were transferred to imprisonment to serve what remained of their sentences. Thus, we use a conservative definition of serving a prison sentence at home under electronic monitoring, and our effect estimates are therefore likely lower bound.

**Figure 2:**
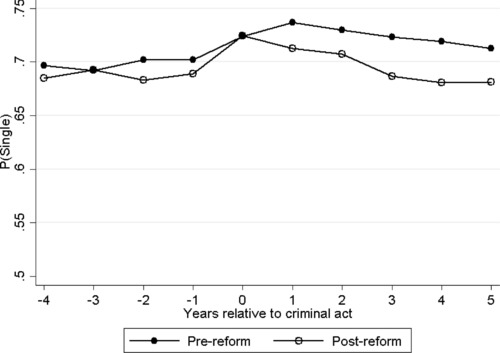
Singlehood Rates for Men Convicted Pre‐ and Postreform (Men Older than 25 Years of Age; Nontraffic Offenses; Sentence Duration Shorter Than or Equal to three Months). *Source*: Own calculations on data from Statistics Denmark.

Figure 2 shows singlehood rate trajectories for the men in the Full Sample, conditional upon whether they were convicted before or after the reform in 2008, which introduced home confinement under electronic monitoring for this offender group. During each of the years before crime and conviction, these men do not differ systematically in their singlehood rates, which fluctuate around 70 percent. Following crime and conviction, those who committed their crime after the reform (and of whom 33.7 percent served their sentence at home under electronic monitoring) have consistently lower singlehood rates than the group who was convicted before the reform.

**Figure 3:**
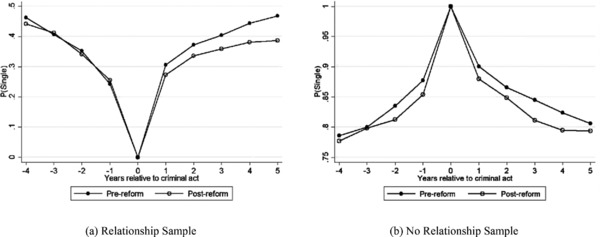
Singlehood Rates for Men Convicted Pre‐ and Postreform (Men Older than 25 Years of Age; Non‐Traffic Offenses; Sentence Duration Shorter than or Equal to three Months), by Sample. *Note*: We advise the reader to note that the figures do not have identical scales on their *y*‐axes. *Source*: Own calculations on data from Statistics Denmark.

Figure 3 shows the singlehood rates for men in the Relationship Sample and the No Relationship Sample. In the Relationship Sample in Figure 3(a), men who were convicted before and after the reform have close to identical singlehood rates before conviction, gradually declining from 50 percent five years before crime and conviction. And again, the singlehood rates of the groups start to gap following conviction, and offenders convicted after the reform (of whom 43.9 percent were home‐confined under electronic monitoring) have consistently lower singlehood rates. Because all offenders in this subsample were in a relationship upon conviction, the difference in their postconviction singlehood rates may be interpreted as a slightly noisy measure of difference in their rate of relationship dissolution. When conditioning on remaining with the same partner as in year 0, this difference is slightly more pronounced (results not show here, but available upon request).

Figure 3(b) shows the singlehood rates for men in the No Relationship Sample. Once again, these men have similar singlehood trajectories leading up to their conviction—at which time they all, by definition, are single. Following crime and conviction, we once again find that offenders convicted after the reform (and of whom 29.8 of offenders served their sentence at home under electronic monitoring) have lower singlehood rates than those convicted before the reform. These lower postconviction singlehood rates for men convicted after the reform could imply that, for men who were single upon conviction, it is easier to find a spouse after having served a sentence under electronic monitoring than after imprisonment.

### Estimation Results

Table 2 shows results from the fixed effects difference‐in‐difference models, which estimate equation (1), across samples. Results from the Full Sample show that men who were convicted after the reform in 2008, and of whom some offenders served their sentence at home under electronic monitoring, had much lower rates of singlehood following conviction than men convicted before the reform, who all served their sentence in prison. The estimated difference is 4.5 percentage points, a substantial difference even when compared to the overall postconviction singlehood rate of around 70 percent. The estimate only appears more substantial if we take into consideration two important points. (1) The estimate is cleansed of unobserved characteristics likely to correlate with treatment status and outcome: first, because of the plausible exogeneity of the reform; second, because we control for year and time effects. Third, because unobserved individual traits that are constant over time are removed by controlling for individual‐level fixed effects. (2) The estimate expresses an ITT estimate, with only around 33.7 percent of the eligible group actually serving their sentence at home under electronic monitoring. Thus, the effect of treatment among those who took up treatment is higher.

**Table 2:** Results from fixed effects difference‐in‐difference models

	(1)	(2)	(3)
	Full Sample	Relationship Sample	No Relationship Sample
Policy effect	−0.045***	–0.091***	–0.028*
	(0.011)	(0.023)	(0.012)
ATT	–0.133	–0.207	–0.094
Fixed effects			
Individual	X	X	X
Year	X	X	X
Time	X	X	X
Prereform treatment status	X	X	X
Control variables	X	X	X
*N* × *T*	45,220	12,470	32,750
*N*	4,522	1,247	3,275

*Notes*: Estimates show the effect of introducing electronic monitoring on singlehood (ITT), by sample. Robust standard errors are in parentheses. We obtain the average treatment effect on the treated (ATT) from the policy effect estimate and the EM participation rate, by sample.

*p < 0.05; **p < 0.01; ***p < 0.001

*Source*: Own calculations on data from Statistics Denmark.

Because we have one‐sided noncompliance in whether or not offenders served time with electronic monitoring, we can calculate the ATT estimate by dividing the ITT with the proportion of offenders convicted after the reform who served under electronic monitoring. The ATT estimate for the Full Sample is 13.3 percentage points, which means that men who served their sentence at home under electronic monitoring experienced remarkably lower singlehood rates following conviction, just as we would expect from the theories outlined above.

Results from the Relationship Sample and the No Relationship Sample only add to our theoretical expectation that electronic monitoring is beneficial to relationship outcomes and could be especially beneficial to offenders who are in a relationship upon conviction. Results show that in the Relationship Sample (middle column of Table 2), postconviction singlehood rates were as much as 9.1 percentage points lower among those convicted after the reform than among those convicted before. This ITT estimate translates into a staggering 20.7 percentage points for those men who actually served their sentence at home under electronic monitoring (the ATT). It is thus safe to say that home confinement under electronic monitoring is very beneficial to couples when compared to imprisonment—their relationships are just much less likely to dissolve.

Results from the No Relationship Sample (last column of Table 2) show that although these men also experienced substantially lower postconviction singlehood rates if convicted after the reform—on average around 3 percentage points—the effect is far smaller than in the Relationship Sample. But still, even for men who were not in a relationship upon conviction, home confinement under electronic monitoring made it easier to find a partner following conviction than was the case for comparable offenders who were imprisoned, an ATT estimate of 9.4 percentage points.

We also estimated equation (1) for a subsample of offenders who were married upon conviction. We did this to see whether nonmarital relationships drive our results, because nonmarital relationships are less formalized and easier to break off. Results (not shown but available on request) reject that hypothesis, and home confinement under electronic monitoring is beneficial to marriage outcomes too. The estimates for marriage are numerically lower than for relationships in general, but this was expected, simply because divorce, for example, takes time. In the Full Sample, rates of nonmarriage are thus almost 10 percentage points lower following conviction among men who served their sentence at home under electronic monitoring than among comparable men who were imprisoned (the ATT).

Importantly, none of the three steps that we apply to analyze the assumptions of our fixed effects difference‐in‐differences model (and which we explicated in the Method section) show signs that the model assumptions are breached. Thus, there is no difference in relationship rates between treated and controls during the years before conviction^8^; not a single covariate predicts whether offenders were convicted before or after the reform once conditioned on other covariates and sentence length; and we find no evidence of population processes driving our main results. For more details on the results from these three steps, we refer the reader to Supporting Information Appendix B.^9^

### Sentence Length

Offenders in our sample serve sentences up to three months in length, and we split the sample by the median sentence length of 40 days (see Figure A1 in Supporting Information Appendix A for a histogram of sentence length in the data). Table 3 reports the results, by sample. For each of the three samples we see results that are remarkably similar to those we presented above. Home confinement under electronic monitoring is just better for relationship outcomes, and sentence length does not seem to play an overly important role in this respect. This result is surprising because we would expect benefits of home confinement under electronic monitoring to correlate with sentence length, simply because longer sentences cause longer separations of spouses, a longer period of absence from society and the courtship market, and a longer period subjected to the negative impacts of imprisonment. In addition, potential spouses could interpret a lengthier sentence as a marker of bad personality. Previous research has generally found a relationship between sentence length and relationship outcomes. However, when we control for individual‐level fixed effect, our results do not align with previous findings. Either this indicates that the correlation between sentence length and relationship outcomes is confounded by offender type, or that offenders in our sample serve sentences of a length so short that we cannot identify changes across duration.

**Table 3:** Results from fixed effects difference‐in‐difference models

	Full Sample		Relationship Sample		No Relationship Sample	
	(1)	(2)	(3)	(4)	(5)	(6)
	<40 days	≥40 days	<40 days	≥40 days	<40 days	≥40 days
Policy effect	–0.046**	–0.046**	–0.095**	–0.081*	–0.031*	–0.028
	(0.015)	(0.016)	(0.031)	(0.033)	(0.016)	(0.017)
ATT	–0.138	–0.135	–0.203	–0.200	–0.110	–0.088
Fixed effects						
Individual	X	X	X	X	X	X
Year	X	X	X	X	X	X
Time	X	X	X	X	X	X
Prereform treatment status	X	X	X	X	X	X
Control variables	X	X	X	X	X	X
*N* × *T*	24,410	20,810	6,620	5,850	17,790	14,960
*N*	2,441	2,081	662	585	1,779	1,496

*Notes*: Estimates show the effect of introducing electronic monitoring on singlehood (ITT), by sample and by sentence length. Robust standard errors are in parentheses. We obtain the average treatment effect on the treated (ATT) from the policy effect estimate and the EM participation rate, by sample.

*p < 0.05; **p < 0.01; ***p < 0.001.

*Source*: Own calculations on data from Statistics Denmark.

## DISCUSSION

In this study, we estimated the effect of imprisonment relative to electronic monitoring on romantic relationship dissolution and on singlehood. Results consistently showed that offenders who serve a prison sentence at home under electronic monitoring had lower singlehood and relationship dissolution rates following conviction, even when we considered unobserved time‐invariant individual characteristics.

In fact, results showed that offenders who serve their prison sentence under electronic monitoring have dramatically better relationship outcomes than comparable offenders who are imprisoned. Male offenders convicted after a penal reform in 2008 that introduced home confinement under electronic monitoring as a noncustodial alternative to imprisonment for offenders older than 25 years of age in Denmark, had on average 4.5 percentage points lower rates of relationship dissolution and singlehood after conviction than comparable offenders convicted before the reform. The group convicted before the reform, who had worse relationship outcomes, all served their sentence in prison. The group convicted after the reform, who had better relationship outcomes, consisted of (on average) similar offenders, 34 percent of them just served their prison sentence at home under electronic monitoring. Being the only difference between the two groups (on average) this means that for those who actually served their prison sentence at home under electronic monitoring, the average effect was even greater: 13.3 percentage points lower risk of relationship dissolution and singlehood.

The theoretical mechanisms that could drive the effect of imprisonment on relationship outcomes imply that imprisonment is likely to have greater impact on the relationship outcomes of men who are in relationships upon conviction. Being a couple, the social stigma of imprisonment might not only blemish the imprisoned man himself, but could also extend to his spouse—leading to a higher risk of relationship dissolution. If a single man becomes imprisoned, this too might blemish him with social stigma. As he seeks a spouse after release, his imprisonment experience is likely to be perceived as an indicator of poor character, and his value on the courtship market decreases. Yet the same is true for those men who were in a relationship upon conviction, but who lost their spouse because of being imprisoned. Our empirical results confirmed this theoretical expectation, and the effect of imprisonment on relationship outcomes is much stronger for men who were in relationships upon conviction than for singles (even though results for singles were substantial too).

Previous research has stressed the importance of duration when studying the impact of incarceration on offenders’ divorce and singlehood rates (Massoglia, Remster, & King, 2011; Siennick, Stewart, & Staff, 2014). None of the offenders in our sample were sentenced to imprisonment for more than three months, but despite these comparatively short sentences, we still found substantial effects of imprisonment on relationship outcomes. To analyze the importance of duration in our specific setup, we also ran analyses by sentence length. We found surprisingly similar results by sentence lengths—for all offenders in our sample as well as by offender type (defined by their relationship status at conviction). Although the theoretical mechanisms that link imprisonment to relationship outcomes are likely to depend on sentence length, we find no such empirical evidence in our sample. This does not mean, however, that sentence length or duration of incarceration is generally uncorrelated with relationship outcomes, especially in other contexts, as this finding could easily just show that results are from Denmark where sentences are generally very short. In the United States, where sentences are so much longer, however, Apel (2016) also reports damaging consequences for relationship outcomes of even very short periods of incarceration.

### Policy Implications

The finding that home confinement under electronic monitoring has a causal effect on relationship outcomes—especially on relationship dissolution—in Denmark has important implications for policy. First, better relationship outcomes could produce better life‐course outcomes in general, and our results show that penal policies can play an important role in securing such better relationship outcomes. Also, the finding that electronic monitoring decreases the singlehood rates even among men who were not in relationships upon conviction is a strong indicator that the future life chances of convicted offenders are improved when they serve their sentence as home confinement rather than in prison. In this way, home confinement under electronic monitoring not only decreases the risk that convicted offenders lose something valuable (their romantic relationship), it also increases their chance of finding something valuable (a partner) in the future—something that could help them remain on the straight and narrow.

Second, even though policies do not translate well across countries, results from the Danish electronic monitoring policies could contribute greatly to policy debates in the United States. To do so, we should explicate the comparability of the two contexts.

#### Sentence Comparability

In Denmark, offenders imprisoned for up to three months (today: six months) might be eligible for home confinement under electronic monitoring. In the United States, sentences are so much longer that any comparison of sentence length seems cumbersome. But jail incarceration in the United States might better compare with imprisonment in Denmark because jail prisoners are often incarcerated for less than one year. Although a significant portion of jail inmates are arrestees and pretrial detainees, whom it might not be feasible to offer home confinement under electronic monitoring, results from Denmark could serve as inspiration for debate about how to handle jail populations in the United States. Adding to the relevance of our results for the United States context, recent research has shown that people who experience even short spells of jail and prison incarceration in the United States suffer immediate and persistent instability in their romantic relationships (Apel, 2016).

#### Offender Comparability

In Denmark, all offenders sentenced to less than three months (today: six months) may apply for home confinement under electronic monitoring, provided specific requirements are met. The Danish Prison and Probation Service decides whether an offender meets those requirements, contingent upon individual characteristics. The Danish Prison and Probation Service places great effort into assuring that only offenders who do not pose a public threat are offered the opportunity to serve their sentence at home under electronic monitoring; it is a time‐consuming process. We do not know whether a similar screening procedure is feasible or even possible in the United States. Thus, debates might benefit from focusing attention either on screening requirements or on offender types already viewed as posing low public threat, such as low‐level drug use and nonviolent offenders.

#### Social Support Comparability

In the United States, probationers and parolees often have to rely on basic social and financial assistance from family and kin. Providing support, according to recent qualitative research, puts strain on those families (Comfort, 2016). But, in Denmark, all citizens are entitled to basic social assistance in case of unemployment or inability to work. This implies that all citizens (even those who are not members of unemployment insurance funds) are entitled to public benefits large enough to support living expenses if they happen to become unemployed. Receiving this basic social assistance does not necessarily alleviate the fact that having a family member on probation or parole strains families and requires social support. Yet, at least the financial burden on those families is smaller in Denmark. Thus, having a family member on electronic monitoring in the United States could be more costly for family and kin than in Denmark.

#### Organizational Comparability

The United States has comparatively high probation and parole rates, which places great workloads on probation and parole officers. Put directly, placing more people under community supervision (such as on electronic monitoring) would probably require significant increases in funding to the community corrections field (DeMichele, 2014). In Denmark, where the workload of community supervision personnel is generally lower, the organizational friction associated with increasing the amount of offenders under electronic monitoring is likely to be lower. This means that the rolling out of a noncustodial alternative to imprisonment, such as home confinement under electronic monitoring, might simply have been easier in Denmark than what we might reasonably expect in the United States.

#### Policy Comparability

Home confinement under electronic monitoring in Denmark is a specific type of punishment, aided by a specific type of technology and implemented in a specific way in a specific context, a way that may or may not be optimal for the United States. Even so, results from Denmark could inspire policymakers in other countries to be attentive to some of the positive aspects of the Danish implementation of electronic monitoring (e.g., Payne, 2014). Electronic monitoring in Denmark, just like any other correctional measure, is a tool applied in certain ways by certain people in certain contexts (DeMichele, 2014). Handpicking from the way this correctional tool is used in Denmark, and in other contexts, could help to assemble electronic monitoring in, for example, the United States, in a way that could improve offender outcomes relative to imprisonment. As we have outlined at length in this paper, home confinement in Denmark constitutes a full package of treatments, which are simply made possible by the technology of electronic monitoring. It is not empirically possible to tell whether better relationship outcomes among offenders who were home‐confined under electronic monitoring came from wearing the GPS tracker, from spending more time with one's family, from adhering to the strict daily routine schedule, or from one of the other elements of home confinement under electronic monitoring in Denmark. Yet by providing details on all of these elements, and by showing that this way of serving a prison sentence produces better relationship outcomes in Denmark, we hope to inspire policymakers in other contexts.

## CONCLUSION

Existing research has shown that, compared to imprisonment, electronic monitoring decreases criminal recidivism (e.g., Di Tella & Schargrodsky, 2013), strengthens the labor market, and improves the educational outcomes of offenders (e.g., Andersen & Andersen, 2014; Larsen, 2016). Also, at least in Denmark, electronic monitoring is cheaper to run than imprisonment. Andersen and Andersen (2014) report that the daily cost of having an offender serve his or her sentence under electronic monitoring is one‐third the cost of having the offender serve it in prison. All in all, this implies additional positive externalities. Now that we also know that electronic monitoring decreases singlehood rates, electronic monitoring seems like an even more viable way to accelerate current decarceration trends—especially among offenders who have pro‐social assets available to them in the form of marriage or a cohabitating partner.

## Figures and Tables

**Figure A1:**
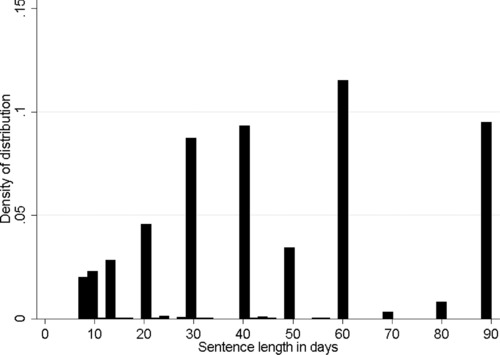
Histogram of Sentence Lengths for Offenders in the Sample. *Source*: Own calculations on data from Statistics Denmark.

**Figure B1:**
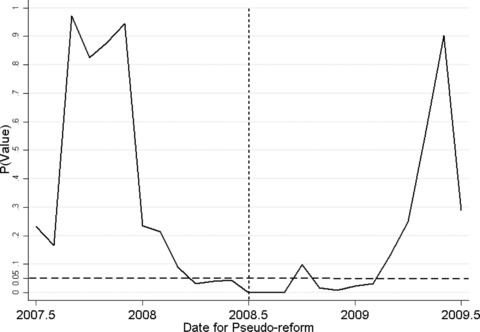
Pseudo‐Test of the Reform, Shifting the Data and Treatment Window ± 1 Year. *Note*: The vertical dashed line indicates the true reform date. The dashed horizontal line indicates *p* = 0.05. *Source*: Own calculations on data from Statistics Denmark.

## Footnotes

1 Danish Corrections Act, Law no. 367: Fuldbyrdelse af straf på bopælen under intensiv overvågning og kontrol samt begrænset fællesskab for “negativt stærke” indsatte m.v. [The execution of sentences as home confinement under intensive monitoring and control, and decreased community privileges for “negatively strong” prisoners, etc.]. Enacted on May 24, 2005.

2 The reform in 2008 prohibited the use of electronic monitoring for offenders convicted on charges of possession of weapons or explosives who were sentenced to a prison term lasting less than two weeks.

3 We do not exploit the other reforms for inference in this study, as these other reforms either targeted offender groups with very low relationship shares, or the reforms were enacted so recently that we do not have sufficient data to perform the required analyses.

4 Also, the men in the sample did not receive prison sentences shorter than two weeks for violating the law on possessing weapons or explosives, nor had they been punished by more than a fine during the previous two years, corresponding to the requirements of the reform.

5 All appendices are available at the end of this article as it appears in JPAM online. Go to the publisher's website and use the search engine to locate the article at http://onlinelibrary.wiley.com.

6 297 female offenders committed crimes that would have included them in our sample from 2006 to 2009. 33 of them served their sentence with electronic monitoring.

7 All appendices are available at the end of this article as it appears in JPAM online. Go to the publisher's website and use the search engine to locate the article at http://onlinelibrary.wiley.com.

8 We tested differences in mean relationship rates among treated and controls for each year before conviction and found no statistically significant differences; see Appendix B. We could also have tested the pre‐conviction linear slopes in relationship rates among treated and controls to emphasize whether they had similar relationship trajectories. But the differences in relationship rates among treated and controls within years before conviction are so meager when compared to the trends for both groups across years that it seems very likely that the slopes of the linear trends prior to conviction are similar too.

9 All appendices are available at the end of this article as it appears in JPAM online. Go to the publisher's website and use the search engine to locate the article at http://onlinelibrary.wiley.com.

## Appendix A

**Table A1:** Results from first stage regression of the reform instrument and covariates on the probability of serving a prison sentence at home under electronic monitoring

	Full Sample	Relationship Sample	No Relationship Sample
Reform	0.339***	0.440***	0.300***
	(0.009)	(0.018)	(0.010)
Age	0.000	0.001	–0.000
	(0.001)	(0.001)	(0.001)
Education	0.012***	0.012***	0.010***
	(0.002)	(0.004)	(0.002)
Theft	0.022	0.099**	0.003
	(0.015)	(0.036)	(0.017)
Violence	0.098***	0.089***	0.098***
	(0.010)	(0.021)	(0.011)
Minority background	–0.004	–0.012	–0.004
	(0.012)	(0.023)	(0.014)
Intercept	–0.192***	–0.230***	–0.156***
	(0.029)	(0.058)	(0.033)
*R* ^2^	0.255	0.333	0.229
*F*‐value for instrument	1,411.79	583.92	855.04
*N*	4,552	1,247	3,275

*Note*: Standard errors are in parentheses.

**p* < 0.05; ***p* < 0.01; ****p* < 0.001.

*Source*: Own calculations on data from Statistics Denmark.

## Appendix B

The pivotal assumption for our analyses is the assumption that treatment and control groups share common trends in singlehood rates, because then it appears feasible that the postconviction differences express the effect of the reform. Because we cannot observe how trends for the treated group would have been absent the reform, the assumption is untestable. Our best strategy is instead to test whether the treatment and control group had similar trends up until they committed their crime, and that the two groups appear balanced on observables. The common trends assumption does not entail that treatment and control groups need have identical singlehood rates, just that they are parallel. However, because, in the subsamples, we condition on a specific relationship status in the year our offenders commit their crimes, it does de facto mean we have to assume identical rates. To test this, we run the following regression:

(B1) $$S\;Single_{it}\sum_{y=-4}^{0}I{\left(Y\right)}_{it}+\psi Policy_{i}+\varphi Policy_{i}\times \sum_{y=-4}^{0}I{\left(Y\right)}_{it}+\varepsilon_{it}$$

for all observations up until the year the offenders committed their crime. Supporting Information B1 reports our findings. Results show that there are no significant or systematic differences in relationship rates across the pre‐ and postreform groups in the years before treatment. Year × Reform = 1 coefficients in the lower half of Supporting Information B1 show the differences between pre‐ and postreform during that year relative to their overall difference, Reform = 1. The coefficients are thus only straightforwardly interpreted when combined. The positive coefficient associated with, for example, Year –4 × Reform = 1 in the No Relationship Sample (last column of Supporting Information B1) does not imply that those convicted after the reform had higher relationship rates than those convicted before the reform (a result that would seem incompatible with the graphical representation in Figure 3b). The difference between the groups should instead be found as the sum of coefficients Reform = 1 + Year –4 × Reform = 1 = –0.022 + 0.013 = –0.009, which resonates better with Figure 3b.

**Table B1:** Testing common trends pretreatment by regressing singlehood on time dummies, reform dummies, and reform‐time interactions

	Full Sample	Relationship Sample	No Relationship Sample
Year relative to crime			
Year –4	–0.005	0.110***	–0.049***
	(0.013)	(0.025)	(0.012)
Year –3	–0.010	0.054*	–0.035**
	(0.013)	(0.025)	(0.012)
Year –2	Ref.	Ref.	Ref.
Year –1	0.000	–0.110***	0.043**
	(0.013)	(0.025)	(0.012)
Year 0	0.022		
	(0.013)		
Reform = 1	–0.019	–0.011	–0.022
	(0.014)	(0.027)	(0.014)
Year interacted with reform status			
Year –4 × Reform = 1	–0.006	–0.011	0.013
	(0.020)	(0.039)	(0.019)
Year –3 × Reform = 1	0.019	0.016	0.021
Year –2 × Reform = 1	(0.020)	(0.039)	(0.019)
	Ref.	Ref.	Ref.
Year –1 × Reform = 1	0.006	0.024	–0.002
	(0.020)	(0.039)	(0.019)
Year 0 × Reform = 1	0.019		
	(0.20)		
Constant	0.702***	0.352***	0.835***
	(0.009)	(0.018)	(0.009)
*F*‐value of Reform dummy and all interactions	0.70	0.26	1.54
*N*	22,610	4,988	13,100

*Notes: We exclude observations for Year = 0 for the two subsamples, because everyone in those samples will either be single or in a relationship that year. Robust standard errors are in parentheses*.

**p* < 0.05; ***p* < 0.01; ****p* < 0.001

*Source*: Own calculations on data from Statistics Denmark.

To further test whether our samples are balanced across observable characteristics beyond what we showed in Table 1 (simple *t*‐tests), we regress the reform dummy on observable characteristics measured at the beginning of the year of the crime and on sentence length. Supporting Information B2 reports the findings. In this conditional model, not a single parameter significantly predicts whether an offender committed his offense before or after the reform. Thus, we conclude that our sample is well balanced.

**Table B2:** Reform dummy regressed on sentence length and covariates for Full Sample and Relationship Sample

	Full Sample	Relationship Sample	No Relationship Sample
Sentence length	–0.000	–0.001	–0.000
	(0.000)	(0.001)	(0.000)
Age	–0.080	–0.060	–0.083
	(0.052)	(0.100)	(0.061)
Age × Age	0.002	0.002	0.002
	(0.001)	(0.003)	(0.002)
Age × Age × Age	–0.000	–0.000	–0.000
	(0.000)	(0.000)	(0.000)
Education	0.003	0.003	0.003
	(0.003)	(0.006)	(0.004)
Theft	0.002	0.056	–0.010
	(0.025)	(0.056)	(0.028)
Violence	–0.025	–0.041	–0.019
	(0.017)	(0.032)	(0.020)
Minority	0.003	–0.011	0.007
	(0.020)	(0.036)	(0.024)
Constant	1.374*	1.090	1.414
	(0.665)	(1.280)	(0.780)
*R* ^2^	0.003	0.011	0.002
*N*	4,522	1,247	3,275

*Note: “Other crime” is the reference category for type of crime. Standard errors are in parentheses*

**p* < 0.05; ***p* < 0.01; ****p* < 0.001

*Source*: Own calculations on data from Statistics Denmark.

Last, to test whether the reform truly caused the differences we observe in relationship rates, or if it is simply a result of an underlying change in the population's relationship patterns, we run a set of pseudo‐regression. We re‐estimate equation (1), but shift the entire data window including the reform date one year in each direction at a monthly increment. Supporting Information B1 reports the *p*‐values for each of these pseudo‐reform estimates. We only find significant effects around the period of the actual reform, which bolster our belief that we are estimating the effect of the reform, not of changing population patterns.
